# Supplementary material for: Modeling glioblastoma heterogeneity as a dynamic network of cell states
Source: Mol Syst Biol. 2021 Sep 16;17(9):e10105. doi: 10.15252/msb.202010105 (PMC8444284; doi:10.15252/msb.202010105)
Supplement: Supplementary file 5 — Source Data for Figure 3 [file MSB-17-e10105-s001.zip › Figure3A_sourcedata/GSEA_3065/nosigdb_state1.GseaPreranked.1622718409535/RICHARDS_VERHAAK_CANCERCELL_2010_PRONEURAL.html]

Details for gene set RICHARDS\_VERHAAK\_CANCERCELL\_2010\_PRONEURAL[GSEA]

|  || Dataset | state1 |
| Phenotype | NoPhenotypeAvailable |
| Upregulated in class | na\_neg |
| GeneSet | RICHARDS\_VERHAAK\_CANCERCELL\_2010\_PRONEURAL |
| Enrichment Score (ES) | -0.6836087 |
| Normalized Enrichment Score (NES) | -2.4418 |
| Nominal p-value | 0.0 |
| FDR q-value | 0.0 |
| FWER p-Value | 0.0 |
Table: GSEA Results Summary

  

Fig 1: Enrichment plot: RICHARDS\_VERHAAK\_CANCERCELL\_2010\_PRONEURAL      
 Profile of the Running ES Score & Positions of GeneSet Members on the Rank Ordered List

  

| PROBE | GENE SYMBOL | GENE\_TITLE | RANK IN GENE LIST | RANK METRIC SCORE | RUNNING ES | CORE ENRICHMENT || 1 | MLLT11 |  |  | 321 | 0.254 | -0.0211 | No |
| 2 | TOX3 |  |  | 586 | 0.188 | -0.0394 | No |
| 3 | STMN1 |  |  | 763 | 0.163 | -0.0498 | No |
| 4 | HMGB3 |  |  | 907 | 0.144 | -0.0578 | No |
| 5 | CNTN1 |  |  | 911 | 0.143 | -0.0515 | No |
| 6 | ICK |  |  | 942 | 0.141 | -0.0480 | No |
| 7 | FERMT1 |  |  | 1163 | 0.117 | -0.0651 | No |
| 8 | CKB |  |  | 1368 | 0.098 | -0.0814 | No |
| 9 | PFN2 |  |  | 1462 | 0.091 | -0.0867 | No |
| 10 | CBX1 |  |  | 1521 | 0.088 | -0.0886 | No |
| 11 | SLCO5A1 |  |  | 1589 | 0.083 | -0.0916 | No |
| 12 | CA10 |  |  | 1674 | 0.077 | -0.0966 | No |
| 13 | SLC1A1 |  |  | 1828 | 0.068 | -0.1091 | No |
| 14 | PAFAH1B3 |  |  | 1834 | 0.068 | -0.1065 | No |
| 15 | BEX1 |  |  | 2118 | 0.054 | -0.1329 | No |
| 16 | GNG4 |  |  | 2285 | 0.048 | -0.1477 | No |
| 17 | ACTR1A |  |  | 2692 | 0.033 | -0.1878 | No |
| 18 | HDAC2 |  |  | 2724 | 0.032 | -0.1894 | No |
| 19 | FHOD3 |  |  | 3786 | 0.008 | -0.2977 | No |
| 20 | CDK5R1 |  |  | 3866 | 0.007 | -0.3055 | No |
| 21 | WASF1 |  |  | 3937 | 0.006 | -0.3124 | No |
| 22 | PPM1E |  |  | 4230 | 0.000 | -0.3423 | No |
| 23 | KDM1A |  |  | 4253 | -0.000 | -0.3445 | No |
| 24 | GADD45G |  |  | 4503 | -0.005 | -0.3698 | No |
| 25 | CDKN1B |  |  | 4655 | -0.007 | -0.3849 | No |
| 26 | RAD21 |  |  | 4916 | -0.011 | -0.4110 | No |
| 27 | VAX2 |  |  | 5250 | -0.016 | -0.4444 | No |
| 28 | TOPBP1 |  |  | 5733 | -0.024 | -0.4926 | No |
| 29 | CDC25A |  |  | 5789 | -0.025 | -0.4971 | No |
| 30 | SATB1 |  |  | 6070 | -0.030 | -0.5244 | No |
| 31 | ZNF510 |  |  | 6123 | -0.031 | -0.5282 | No |
| 32 | PAK3 |  |  | 6174 | -0.032 | -0.5318 | No |
| 33 | RAP2A |  |  | 6372 | -0.036 | -0.5503 | No |
| 34 | DPYSL4 |  |  | 6529 | -0.039 | -0.5645 | No |
| 35 | PPM1D |  |  | 6609 | -0.041 | -0.5707 | No |
| 36 | ZNF184 |  |  | 6666 | -0.042 | -0.5745 | No |
| 37 | AMOTL2 |  |  | 6753 | -0.044 | -0.5812 | No |
| 38 | CDC7 |  |  | 6788 | -0.045 | -0.5826 | No |
| 39 | MARCKSL1 |  |  | 6861 | -0.046 | -0.5879 | No |
| 40 | REEP1 |  |  | 6897 | -0.047 | -0.5892 | No |
| 41 | MVB12B |  |  | 7012 | -0.050 | -0.5986 | No |
| 42 | E2F3 |  |  | 7064 | -0.052 | -0.6014 | No |
| 43 | VEZF1 |  |  | 7182 | -0.055 | -0.6108 | No |
| 44 | FAM110B |  |  | 7201 | -0.055 | -0.6101 | No |
| 45 | BCOR |  |  | 7226 | -0.056 | -0.6099 | No |
| 46 | DPF1 |  |  | 7301 | -0.058 | -0.6148 | No |
| 47 | GSK3B |  |  | 7351 | -0.059 | -0.6171 | No |
| 48 | RBPJ |  |  | 7588 | -0.066 | -0.6382 | No |
| 49 | RALGPS2 |  |  | 7602 | -0.066 | -0.6364 | No |
| 50 | ATAT1 |  |  | 7623 | -0.067 | -0.6354 | No |
| 51 | ZNF821 |  |  | 7903 | -0.078 | -0.6604 | No |
| 52 | SEC61A2 |  |  | 7931 | -0.079 | -0.6595 | No |
| 53 | ZNF248 |  |  | 7957 | -0.080 | -0.6583 | No |
| 54 | PDE10A |  |  | 7959 | -0.080 | -0.6547 | No |
| 55 | CAMSAP2 |  |  | 8070 | -0.084 | -0.6621 | No |
| 56 | RNFT2 |  |  | 8084 | -0.084 | -0.6595 | No |
| 57 | KAT7 |  |  | 8089 | -0.085 | -0.6560 | No |
| 58 | BCL7A |  |  | 8103 | -0.085 | -0.6534 | No |
| 59 | FBXO21 |  |  | 8130 | -0.086 | -0.6520 | No |
| 60 | GSTA4 |  |  | 8147 | -0.087 | -0.6496 | No |
| 61 | ARHGAP33 |  |  | 8204 | -0.089 | -0.6512 | No |
| 62 | EPB41 |  |  | 8232 | -0.091 | -0.6497 | No |
| 63 | MCM10 |  |  | 8339 | -0.096 | -0.6561 | No |
| 64 | DBN1 |  |  | 8449 | -0.102 | -0.6626 | No |
| 65 | CASK |  |  | 8532 | -0.107 | -0.6660 | No |
| 66 | DNM3 |  |  | 8701 | -0.117 | -0.6777 | Yes |
| 67 | DCAF7 |  |  | 8714 | -0.119 | -0.6734 | Yes |
| 68 | CSNK1E |  |  | 8730 | -0.120 | -0.6694 | Yes |
| 69 | MAPT |  |  | 8749 | -0.121 | -0.6656 | Yes |
| 70 | CRMP1 |  |  | 8801 | -0.125 | -0.6651 | Yes |
| 71 | YPEL1 |  |  | 8826 | -0.126 | -0.6616 | Yes |
| 72 | ATAD5 |  |  | 9000 | -0.142 | -0.6727 | Yes |
| 73 | MMP15 |  |  | 9003 | -0.143 | -0.6663 | Yes |
| 74 | TTC3 |  |  | 9024 | -0.144 | -0.6617 | Yes |
| 75 | TMSB15A |  |  | 9079 | -0.150 | -0.6602 | Yes |
| 76 | TMCC1 |  |  | 9091 | -0.151 | -0.6543 | Yes |
| 77 | ARHGEF9 |  |  | 9378 | -0.196 | -0.6745 | Yes |
| 78 | CLASP2 |  |  | 9383 | -0.197 | -0.6658 | Yes |
| 79 | TOP2B |  |  | 9444 | -0.211 | -0.6621 | Yes |
| 80 | PHLPP1 |  |  | 9463 | -0.217 | -0.6539 | Yes |
| 81 | NLGN3 |  |  | 9554 | -0.241 | -0.6519 | Yes |
| 82 | ERBB3 |  |  | 9601 | -0.258 | -0.6446 | Yes |
| 83 | DPP6 |  |  | 9606 | -0.262 | -0.6328 | Yes |
| 84 | SEZ6L |  |  | 9628 | -0.272 | -0.6224 | Yes |
| 85 | FXYD6 |  |  | 9631 | -0.273 | -0.6099 | Yes |
| 86 | ABAT |  |  | 9646 | -0.278 | -0.5985 | Yes |
| 87 | LRP6 |  |  | 9656 | -0.286 | -0.5861 | Yes |
| 88 | DLL3 |  |  | 9678 | -0.297 | -0.5745 | Yes |
| 89 | SOX2 |  |  | 9680 | -0.298 | -0.5607 | Yes |
| 90 | PODXL2 |  |  | 9686 | -0.301 | -0.5473 | Yes |
| 91 | SRGAP3 |  |  | 9689 | -0.304 | -0.5334 | Yes |
| 92 | SOX4 |  |  | 9704 | -0.318 | -0.5200 | Yes |
| 93 | ADGRL3 |  |  | 9711 | -0.324 | -0.5056 | Yes |
| 94 | TSPAN3 |  |  | 9713 | -0.326 | -0.4905 | Yes |
| 95 | CSPG5 |  |  | 9721 | -0.330 | -0.4759 | Yes |
| 96 | MMP16 |  |  | 9724 | -0.335 | -0.4605 | Yes |
| 97 | MYO10 |  |  | 9725 | -0.335 | -0.4450 | Yes |
| 98 | SOX11 |  |  | 9730 | -0.339 | -0.4296 | Yes |
| 99 | MTSS1 |  |  | 9741 | -0.350 | -0.4144 | Yes |
| 100 | ZEB2 |  |  | 9745 | -0.353 | -0.3983 | Yes |
| 101 | P2RX7 |  |  | 9755 | -0.360 | -0.3825 | Yes |
| 102 | MARCKS |  |  | 9763 | -0.370 | -0.3660 | Yes |
| 103 | RUFY3 |  |  | 9765 | -0.372 | -0.3489 | Yes |
| 104 | CHD7 |  |  | 9769 | -0.379 | -0.3316 | Yes |
| 105 | OLIG2 |  |  | 9776 | -0.386 | -0.3143 | Yes |
| 106 | C1orf61 |  |  | 9813 | -0.479 | -0.2957 | Yes |
| 107 | ALCAM |  |  | 9818 | -0.493 | -0.2732 | Yes |
| 108 | GPM6A |  |  | 9820 | -0.499 | -0.2502 | Yes |
| 109 | GRIA2 |  |  | 9846 | -0.638 | -0.2231 | Yes |
| 110 | MAP2 |  |  | 9857 | -0.735 | -0.1900 | Yes |
| 111 | NCAM1 |  |  | 9862 | -0.751 | -0.1555 | Yes |
| 112 | TTYH1 |  |  | 9872 | -1.033 | -0.1084 | Yes |
| 113 | C1QL1 |  |  | 9876 | -1.171 | -0.0543 | Yes |
| 114 | BCAN |  |  | 9878 | -1.175 | 0.0001 | Yes |
Table: GSEA details [plain text format]

  

Fig 2: RICHARDS\_VERHAAK\_CANCERCELL\_2010\_PRONEURAL: Random ES distribution      
 Gene set null distribution of ES for **RICHARDS\_VERHAAK\_CANCERCELL\_2010\_PRONEURAL**
